# Errata

**DOI:** 10.4269/ajtmh.2010.833err

**Published:** 2010-09

In *Am J Trop Med Hyg 83:*56–60, one of the author's names appears incorrectly. The correct name is Panagiotis Karanis, not Karanis Panagiotis. The journal regrets the error.

In *Am J Trop Med Hyg 83:*174–182, there were errors in Table 1. The corrected table appears below. The journal regrets these errors.

Correction: Results, “Overall Incidence”, 3rd paragraph, last sentence: “With the exception of the Pacific and Middle Atlantic regions, incidence in all regions increased more than 100% from the first half (2000–2003) to the second half (2004–2007) of the reporting period (Table 1).”

## Figures and Tables

**Table 1:** Rocky Mountain spotted fever case reports and incidence (per million persons) by state and geographic region, 2000–2007, United States (National Electronic Telecommunications System for Surveillance)

Region	2000–2007		2000–2003	2004–2007	Percent change 2000–2003 vs. 2004–2007
	n	Incidence	Incidence	Incidence	
New England	95	0.84	0.46	1.21	+163%
Connecticut	6	0.22	0.22	0.22	
Maine*	1	0.10	0.00	0.19	
Massachusetts	58	1.13	0.62	1.63	
New Hampshire	4	0.39	0.20	0.58	
Rhode Island	25	2.94	1.41	4.47	
Vermont	1	0.20	0.00	0.40	
Middle Atlantic	489	1.52	1.11	1.93	+74%
New Jersey	170	2.48	1.56	3.39	
New York	127	0.83	0.48	1.16	
Pennsylvania	192	1.95	1.77	2.12	
East North Central	298	0.81	0.53	1.10	+108%
Illinois	124	1.23	0.68	1.77	
Indiana	30	0.60	0.45	0.76	
Michigan	30	0.37	0.30	0.45	
Ohio	107	1.17	0.86	1.48	
Wisconsin	7	0.16	0.00	0.32	
West North Central	1146	7.30	3.79	10.74	+184%
Iowa	40	1.70	0.77	2.62	
Kansas	22	1.01	0.37	1.64	
Minnesota	21	0.52	0.20	0.83	
Missouri	963	21.00	11.09	30.64	
Nebraska	74	5.32	2.18	8.40	
North Dakota	2	0.39	0.39	0.39	
South Dakota	24	3.88	3.29	4.46	
South Atlantic	5704	13.01	8.05	17.67	+120%
Delaware	67	10.19	4.69	15.40	
Dist. Columbia	10	2.15	1.73	2.57	
Florida	130	0.95	0.80	1.08	
Georgia	389	5.49	3.30	7.52	
Maryland	514	11.69	9.57	13.74	
North Carolina	3581	52.61	29.70	74.16	
South Carolina	445	13.28	12.49	14.03	
Virginia	529	8.93	4.29	13.35	
West Virginia	39	2.70	1.67	3.74	
East South Central	1525	10.94	6.81	14.95	+120%
Alabama	381	10.55	3.92	17.03	
Kentucky	28	0.85	0.86	0.84	
Mississippi	156	6.77	7.18	6.38	
Tennessee	960	20.33	13.07	27.29	
West South Central	2063	7.81	4.92	10.56	+115%
Arkansas	802	36.57	23.27	49.44	
Louisiana	25	0.70	0.28	1.14	
Oklahoma	1064	37.85	24.68	50.71	
Texas	172	0.96	0.40	1.49	
Mountain	190	1.20	0.62	1.74	+179%
Arizona	51	1.11	0.09	2.02	
Colorado	22	0.60	0.34	0.85	
Idaho	29	2.61	0.75	4.33	
Montana	14	1.89	1.93	1.86	
Nevada	7	0.38	0.82	0.00	
New Mexico	24	1.59	0.54	2.60	
Utah	6	0.31	0.54	0.10	
Wyoming	37	9.19	6.05	12.23	
Pacific	21	0.06	0.06	0.05	–
Alaska*	0	–	–	–	
California	7	0.02	0.04	0.01	
Hawaii*	0	–	–	–	
Oregon	14	0.49	0.43	0.55	
Washington*	0	–	–	–	
Total	11,531	4.94	3.02	6.79	125%

* RMSF not considered a notifiable disease for all study years

